# Diagnosis of Exclusion: A Case Report of Probable Glatiramer Acetate-Induced Eosinophilic Myocarditis

**DOI:** 10.1155/2014/786342

**Published:** 2014-07-03

**Authors:** Christopher J. Michaud, Heather M. Bockheim, Muhammad Nabeel, Timothy E. Daum

**Affiliations:** ^1^Department of Pharmacy, Spectrum Health, 100 Michigan Street NE, MC 001, Grand Rapids, MI 49503, USA; ^2^Internal Medicine, Spectrum Health, Grand Rapids, MI 49503, USA; ^3^Pulmonary Critical Care, Spectrum Health Medical Group, Grand Rapids, MI 49503, USA

## Abstract

*Importance.* Medication-induced eosinophilia is an acknowledged, often self-limiting occurrence. Glatiramer acetate, a biologic injection used in the management of relapsing-remitting multiple sclerosis, is widely regarded as a safe and effective medication and lists eosinophilia as an infrequent side effect in its package insert. Contrary to reports of transient, benign drug-induced eosinophilia, we describe a case of probable glatiramer acetate-induced eosinophilia that ultimately culminated in respiratory distress, shock, and eosinophilic myocarditis. *Observations.* A 59-year-old female was admitted to the hospital after routine outpatient labs revealed leukocytosis (43,000 cells/mm^3^) with pronounced hypereosinophilia (63%). This patient had been using glatiramer acetate without complication for over 10 years prior to admission. Leukocytosis and hypereosinophilia persisted as a myriad of diagnostic evaluations returned negative, ultimately leading to respiratory depression, shock, and myocarditis. Glatiramer acetate was held for the first time on day 6 of the hospital stay with subsequent resolution of leukocytosis, hypereosinophilia, respiratory distress, and shock. *Conclusions and Relevance.* Glatiramer acetate was probably the cause of this observed hypereosinophilia and the resulting complications. Reports of glatiramer-induced eosinophilia are rare, and few case reports regarding medication-induced hypereosinophilia describe the severe systemic manifestations seen in this patient.

## 1. Introduction

In 1996, the United States FDA approved glatiramer acetate (Copaxone) for the management of relapsing-remitting multiple sclerosis (RRMS). More than 15 years later, it retains an important place in RRMS therapy due to its nonimmunosuppressant mechanism of action, sustained efficacy data, and considerable safety and tolerability profiles. Notably, the more severe and intolerable adverse effects commonly attributed to other RRMS therapies (e.g., leucopenia, flu-like symptoms, thyroid disease, and alopecia) have been seen rarely or not at all with glatiramer acetate [1–4]. Still, no medication is void of potential risk, and serious adverse events (including death) have been reported in patients receiving glatiramer acetate [5].

One such reported adverse reaction is the development of eosinophilia. Eosinophilia is listed as an “infrequent” adverse effect in the Copaxone package insert, and it has only been consistently shown to occur in a dose-dependent fashion in rat and monkey models [4, 6]. The five largest prospective, randomized controlled trials comparing glatiramer acetate to placebo, however, reported no cases of eosinophilia, and scarce reports of glatiramer-induced eosinophilia describe transient and benign phenomena [7–12]. We encountered a case of a patient who, after 10 years of stable glatiramer acetate (GA) therapy, developed a probable glatiramer-induced hypereosinophilic syndrome, ultimately manifesting as shock and eosinophilic myocarditis.

## 2. Case Description

A 59-year-old Caucasian female weighing 94 kg presented to the emergency room with a 2-week history of progressive weakness and fatigue. Her medical history included relapsing-remitting multiple sclerosis (diagnosed in 1998), asthma, depression, and tenosynovitis. Since suffering “a few” major RRMS attacks in the first 3 years after diagnosis, she described her subsequent disease course as “attack-free,” consisting of a slow, progressive decline without major symptoms. The month before admission she had been shopping, driving, and participating in all normal daily activities. She had a 40-pack-year history of smoking but reportedly quit 5 years prior to admission and denied alcohol and illicit drug use. Her home medications included aspirin (81 mg daily), citalopram (20 mg daily), docusate sodium (100 mg daily), GA (20 mg subcutaneously daily since 2001), loratadine (10 mg daily), calcium with vitamin D (1 tablet twice daily), fluticasone/salmeterol diskus 250/50 (1 puff twice daily), albuterol MDI (as needed), and a daily multivitamin. She remarked on admission that her medication compliance was normally very high, but she had been “missing many doses” of her enteral medications since her symptoms began 2 weeks prior. She reported that no doses of GA were missed during this time. The patient had no known drug or food allergies.

The patient was initially seen outpatient by her primary care physician. When routine lab work revealed a white blood cell count of 43,000 cells/mm^3^, she was referred to the emergency department. Her initial vital signs were stable: temperature, 36.4°C; heart rate, 108 beats/min; respiratory rate, 18 breaths/min; blood pressure, 124/70 mm Hg; and oxygen saturation, 92% on room air. The physical examination was grossly normal, and a chest X-ray was negative for acute pulmonary processes. Serum sodium, potassium, chloride, bicarbonate, urea nitrogen, and glucose were all within normal limits; serum creatinine was 0.66 mg/dL, and CRP was 110 mg/L (normal 0–10). A complete blood count (CBC) confirmed outpatient laboratory studies: hemoglobin, 13.5 g/dL; hematocrit, 38.5%; platelets, 467,000 cells/mcL; and white blood cell count, 46,000 cells/mm^3^, and a differential showed 24% segs (normal: 35–80), 4% bands (0–10), 5% lymphocytes (20–50), 3% monocytes (2–12), 1% basophils (0–2), and 63% eosinophils (0–6). Coagulation studies, total bilirubin, and alkaline phosphatase were normal, with slight increases in alanine aminotransferase, aspartate aminotransferase (both 72 IU/L), and lactate dehydrogenase (617 U/L). Troponin T was elevated at 1.160 ng/mL (normal high < 0.030 ng/mL) and an echocardiogram reported an ejection fraction of 50% with mild left ventricular hypertrophy but no wall motion abnormalities. Blood and urine cultures were obtained, the patient was given a dose of ceftriaxone, all home medications were continued, and the patient was admitted to the internal medicine service for further diagnostic workup.

On day 1 of the patient's hospital stay, hematology/oncology, infectious disease, and neurology subspecialists were consulted. Fluorescent in situ hybridization (FISH) studies were negative, bone marrow biopsy was normal save a marked eosinophilia (40.91 K/mcL), serum protein electrophoresis (SPEP) showed an acute phase reaction pattern (decreased albumin and increased alpha-1 and alpha-2 globulins) and nonspecific beta-gamma bridging, and CT scans of the chest, abdomen, and pelvis were negative for infection and malignancy. The patient had no recent or remote history of travel beyond the Midwestern United States, where parasitic infection is extremely uncommon. Strongyloides and coccidioidomycosis antibody panels were negative, as were Hepatitis A, B, and C antibodies. Two sets of blood cultures were negative, and a urine culture supported a diagnosis of asymptomatic bacteriuria, growing only coagulase-negative staphylococcus. MRI of the brain with and without contrast revealed extensive white matter abnormalities consistent with her diagnosis but showed no evidence of abnormal enhancement to indicate acute activity; it was considered generally stable compared to her most recent MRI, performed in 2009. It was acknowledged that GA could cause eosinophilia in rare instances, but, with diagnostic studies pending and considering this patient's 10+ year of history of successful use, it was favored to continue GA therapy.

During days 2–4, folate, cortisol, and TSH checks were normal and Aspergillus antibody, ANA, c-ANCA, and p-ANCA tests were negative. Troponins were trended and remained between 1.30 and 1.50 ng/mL. The patient remained afebrile, displayed normal vital signs without additional medication, and maintained oxygen saturations > 92% on room air with intermittent nasal cannula support during this time. Leukocytosis and eosinophilia did persist and continued to worsen (Table 1).

On day 5, the patient became suddenly hypoxemic requiring BiPAP (FiO_2_ 70%), tachycardic, tachypneic, hypotensive, and febrile and was transferred to the intensive care unit. A chest X-ray showed the development of diffuse bilateral opacities. An arterial blood gas demonstrated a metabolic lactic acidosis, with pH 7.25, pCO_2_ 32, PO_2_ 117, and HCO_3_ 14 (lactate 4.2 mmol/L). The hypoxemic respiratory failure and shock were thought secondary to sepsis, and the patient was started on intravenous norepinephrine, broad-spectrum antibiotics, furosemide, methylprednisolone, and other standard early goal-directed therapies. A serum IgE level was found to be 897 IU/mL (normal 0–180). Troponin T was elevated at 2.300 ng/mL, and a repeat echocardiogram now reported a reduced ejection fraction of 25% (Table 1). Her cardiogenic shock progressed requiring the addition of vasopressin and milrinone. As her clinical condition continued to worsen and in the absence of any positive diagnostics, the decision was made to discontinue the daily GA injections (last dose received was day 5 at 0840).

By the morning of day 7, the patient's white blood cell count and eosinophilia had markedly decreased (Table 1), and she maintained oxygen saturation >92% on high-flow nasal cannula (25 L/min). However, a second echocardiogram and a cardiac MRI confirmed a reduced ejection fraction of 21%. This prompted an endomyocardial biopsy that revealed focal myocarditis with prominent eosinophilic infiltrates.

By day 8, leukocytosis had continued decreasing towards normal and eosinophilia had completely resolved. Intravenous steroids and inotropic agents, as well as intermittent vasopressors, were continued for myocarditis management in the subsequent days. She continued to improve and was transferred out of the intensive care unit on day 12. On day 13, GA was reinitiated at the same dose, once daily. It was felt that, despite the recent events, a relapsing episode of RRMS could be potentially devastating and GA had controlled her disease for many years prior to admission. She was monitored as an inpatient for 3 days with normal blood counts and no evidence of eosinophilia and was discharged to home with healthcare assistance on day 15.

In the 30 days after discharge, the patient continued a prolonged prednisone taper and reported no additional adverse events related to GA; during this time, a repeat CBC showed no leukocytosis and no eosinophilia. Fourteen days after steroids were stopped, however, a CBC revealed 14% eosinophils. One month later, the patient again reported progressive weakness and fatigue and a CBC showed 15% lymphocytes, 12% monocytes, and 23% eosinophils. She was placed back on prednisone and referred to an immunologist to consider alternative RRMS therapies.

## 3. Discussion

Glatiramer acetate is a mixture of 4 amino acid polymers that, together, are antigenically similar to myelin basic protein. T-lymphocyte suppressor cell modulation is thought to be its primary mechanism, thus preventing relapses of RRMS [4]. Given the alternative agents available to treat this disease and their associated adverse effects, GA has been widely regarded as a safe and effective therapy [7, 14]. The most commonly reported side effects include rash (19%), nausea (15%), dyspnea (14%), chest pain (13%), anxiety (13%), infection (30%), and injection site reactions (27–49%) [4].

To our knowledge, this is the first published account of a probable glatiramer-induced hypereosinophilia, particularly regarding the severity, persistence, and end-organ effects of the reaction. We searched MEDLINE and PubMed for prospective, randomized GA trials that assessed adverse effects and for case reports relating GA to eosinophilia using the terms “glatiramer acetate,” “Copaxone,” “safety,” “tolerability,” “eosinophilia,” and “hypereosinophilic syndrome.” We uncovered a study by Ramot et al. linking GA to eosinophilia in an animal model but, otherwise, found no evidence of a connection [6]. One Internet report, connected to the FDA adverse events reporting database, stated that, of 9,277 patients who reported to have side effects while taking Copaxone, 3 experienced eosinophilia (0.03%) [15]. Teva Pharmaceuticals was contacted and was unable to provide additional information regarding eosinophilia's designation as an “infrequent” side effect in the package insert.

It is important to note that many other medications have been implicated in causing a hypereosinophilic hypersensitivity response. The syndrome, termed drug reaction with eosinophilia and systemic symptoms (DRESS), has been described in case reports and systematic reviews (Table 2) [13, 16]. While components of the case described here are in line with this syndrome's description (marked eosinophilia, progressive heart and lung dysfunction, and improvement upon drug discontinuation), there were also classic signs of DRESS not present (no rash, fever, or hepatitis; atypical time of onset) [13, 16, 17]. The degree of systemic complication seen in this case is also rarely described in the classic DRESS syndrome.

According to the Naranjo Adverse Drug Reaction Probability Scale, GA was probably the cause of this patient's hypereosinophilia and resulting sequelae (score 5-6) [18]. Support for causality is found in the myriad of other possible causes that were ruled out during the course of her hospital stay, the rapid reversal of shock, respiratory distress, and eosinophilia after drug discontinuation, and the recurrence of eosinophilia when steroids were stopped and GA remained. Additionally, no drug-drug or drug-food interactions were apparent that would have acutely altered the pharmacokinetics or pharmacodynamics of the injection. However, this patient was stable on GA for over 10 years prior to admission, other classic drug-hypersensitivity symptoms were not present, and high-dose steroids were initiated at the time of GA discontinuation. Regarding her postdischarge course, steroids were effective at quelling the hypereosinophilia in the presence of GA; however, it recurred when the prednisone was discontinued.

Serious medication-induced adverse events are often diagnoses of exclusion. Given the diagnostic workup and timeline of events experienced by this patient, there exists the possibility that GA therapy induced this hypereosinophilic syndrome, which ultimately culminated in respiratory distress, shock, and eosinophilic myocarditis.

## 4. Conclusion

We propose that glatiramer acetate may have contributed to this patient's hypereosinophilic syndrome, though evidence to establish causality is not definitive. Reports of glatiramer-induced eosinophilia are rare, and few case reports regarding medication-induced hypereosinophilia describe the severe systemic manifestations seen in this patient. Healthcare providers are encouraged to include medication-induced abnormalities in the differential diagnosis when treating patients with hypereosinophilia.

## Figures and Tables

**Table 1:** Time course of medication administration and laboratory data.

Test	Day 0	Day 1	Day 2	Day 3	Day 4	Day 5	Day 6	Day 7	Day 8
WBC (×10^3^/mcL)	45.25	43.05	48.36	52.73	58.23	63.47	77.06	36.96	29.36
Eosinophils (%)	63	54	61	64	—	69	66	20	3
Troponin T (ng/mL)	—	1.360	1.550	1.540	—	—	2.300	—	1.740
LVEF (%)	50	—	—	—	—	—	25	21	—
GA dose (mg)	20	20	20	20	20	20	—	—	—
Methylprednisolone dose (mg)	—	—	—	—	—	—	125	1000	1000

WBC: white blood cell count; LVEF: left ventricular ejection fraction; and GA: glatiramer acetate.

**Table 2:** Published case reports related to drug reaction with eosinophilia and systemic symptoms (DRESS).

Medication∗	Published cases
Abacavir	5
Allopurinol	19
Carbamazepine	47
Dapsone	4
Lamotrigine	10
Mexiletine	5
Minocycline	3
Nevirapine	8
Phenobarbital	10
Phenytoin	7
Sulfasalazine	4
Vancomycin	4

*32 additional medications have been described in ≤2 case reports each.

Adapted with permission from Cacoub et al. [13].

## References

[1] Kieseier BC, Stüve O (2011). A critical appraisal of treatment decisions in multiple sclerosis-old versus new. *Nature Reviews Neurology*.

[2] Plosker GL (2011). Interferon-*β*-1b: a review of its use in multiple sclerosis. *CNS Drugs*.

[3] Cohen JA, Barkhof F, Comi G (2010). Oral fingolimod or intramuscular interferon for relapsing multiple sclerosis. *The New England Journal of Medicine*.

[4] *Copaxone [package insert]*.

[5] Khan O, Rieckmann P, Boyko A, Selmaj K, Zivadinov R (2013). Three times weekly glatiramer acetate in relapsing-remitting multiple sclerosis. *Annals of Neurology*.

[6] Ramot Y, Rosenstock M, Klinger E, Bursztyn D, Nyska A, Shinar DM (2012). Comparative long-term preclinical safety evaluation of two glatiramoid compounds (glatiramer Acetate, Copaxone, and TV-5010, protiramer) in rats and monkeys. *Toxicologic Pathology*.

[7] Wolinsky JS, Narayana PA, O'Connor P (2007). Glatiramer acetate in primary progressive multiple sclerosis: results of a multinational, multicenter, double-blind, placebo-controlled trial. *Annals of Neurology*.

[8] Comi G, Filippi M, Wolinsky JS (2001). European/Canadian multicenter, double-blind, randomized, placebo-controlled study of the effects of glatiramer acetate on magnetic resonance imaging-measured disease activity and burden in patients with relapsing multiple sclerosis. *Annals of Neurology*.

[9] Bornstein MB, Miller A, Slagle S (1987). A pilot trial of cop 1 in exacerbating-remitting multiple sclerosis. *The New England Journal of Medicine*.

[10] Sindic CJM, Seeldrayers P, Vande Gaer L (2005). Long-term follow up of glatiramer acetate compassionate use in Belgium. *Acta Neurologica Belgica*.

[11] Johnson KP, Brooks BR, Cohen JA (1998). Extended use of glatiramer acetate (Copaxone) is well tolerated and maintains its clinical effect on multiple sclerosis relapse rate and degree of disability. *Neurology*.

[12] La Mantia L, Munari LM, Lovati R (2010). Glatiramer acetate for multiple sclerosis. *Cochrane Database of Systematic Reviews*.

[13] Cacoub P, Musette P, Descamps V (2011). The DRESS syndrome: a literature review. *The American Journal of Medicine*.

[14] Cohen JA, Rovaris M, Goodman AD, Ladkani D, Wynn D, Filippi M (2007). Randomized, double-blind, dose-comparison study of glatiramer acetate in relapsing-remitting MS. *Neurology*.

[15] From FDA Reports: Copaxone and Eosinophilia. http://www.ehealthme.com/ds/copaxone/eosinophilia.

[16] Walsh SA, Creamer D (2011). Drug reaction with eosinophilia and systemic symptoms (DRESS): a clinical update and review of current thinking. *Clinical and Experimental Dermatology*.

[17] Sullivan JR, Shear NH (2001). The drug hypersensitivity syndrome: what is the pathogenesis?. *Archives of Dermatology*.

[18] Naranjo CA, Busto U, Sellers EM (1981). A method for estimating the probability of adverse drug reactions. *Clinical Pharmacology and Therapeutics*.

